# Effects of Different Shaped Nanoparticles on the Performance of Engine-Oil and Kerosene-Oil: A generalized Brinkman-Type Fluid model with Non-Singular Kernel

**DOI:** 10.1038/s41598-018-33547-z

**Published:** 2018-10-16

**Authors:** Farhad Ali, Aamina, Ilyas Khan, Nadeem Ahmad Sheikh, Madeha Gohar, I. Tlili

**Affiliations:** 1 Computational Analysis Research Group, Ton Duc Thang University, Ho Chi Minh City, Vietnam; 2 Faculty of Mathematics and Statistics, Ton Duc Thang University, Ho Chi Minh City, Vietnam; 3 Department of Mathematics, City University of Science and Information Technology, Peshawar, Khyber Pakhtunkhwa Pakistan; 4 Energy and Thermal Systems Laboratory, National Engineering School of Monastir, Street Ibn El Jazzar, 5019 Monastir, Tunisia

## Abstract

In the modern era, diathermic oils have been gotten the great attention from researchers due to its notable and momentous applications in engineering, mechanics and in the industrial field. The aim of this paper is to model the problem to augment the heat transfer rate of diathermic oils, specifically, Engine-oil (EO) and Kerosene-oil (KO) are taken. The present work is dedicated to examine the shape impacts of molybdenum-disulfide (MoS_2_) nanoparticles in the free convection magnetohydrodynamic (MHD) flow of Brinkman-type nanofluid in a rotating frame. The problem is modeled in terms of partial differential equations with oscillatory boundary conditions. The integer-order model is transformed to fractional-order model in time (Caputo-Fabrizio). The exact solutions are obtained using the Laplace transform technique. Figures are drawn to compare the different non-spherically shaped molybdenum-disulfide nanoparticles on secondary and primary velocities. The Nusselt number is computed in the tabular form and discussed in detail. It is worth noting that platelet and blade shape of MoS_2_ nanoparticle has more tendency to improve the heat transfer rate of both fluids as compared to nanoparticles with brick and cylinder shapes. It is also shown that the rate of heat transfer enhances 13.51% by adding MoS_2_ in engine oil which improved its lubrication properties.

## Introduction

Convective heat transfer in nanofluids is a topic of major contemporary interest both in sciences and in engineering. Heating or cooling fluids such as water, ethylene glycol and engine oil plays a crucial role in the thermal management of high-tech industries such as improvement of the thermal performance of cooling system together with the reduction of their required surface area has always been a great technical challenge. Research carried out on this subject can be classified into three general approaches: finding the best geometry for cooling deceives, decreasing the characteristic length and recently increasing the thermal performance of the coolant. The latest approach is based on the discovery of nanofluids^1^.

Nanofluid is a novel heat-transfer fluid prepared by dispersing nanometer-sized solid particles of metal, carbides, and nitrides in the traditional heat-transfer fluid to increase thermal conductivity and heat-transfer performance. Nanofluid was coined by Choi in 1995 at Argonne National Laboratory of the USA. Around the last few decades, many researchers have attracted towards nanofluid due to their high thermal performance as compared to the base fluid. Sundar *et al*.^2^ investigated experimentally the effect of *Al*_2_*O*_3_ and *CuO* on the mixture of ethylene glycol (EG) and water (*H*_2_*O*), which is considered as a base fluid. They observed an enhancement of 9.8% to 17.89% in thermal conductivity for *Al*_2_*O*_3_ nanofluid and 15.6% to 24.56% for CuO nanofluid, under the temperature range from 15 °C to 50 °C at 0.8% volume concentration compared to the base fluid respectively. This study eventuated that CuO has more tendency to improve the thermal conductivity as compared to *Al*_2_*O*_3_. A number of interesting studies have been listed regarding the improvement of thermal conductivity in^3–6^. Nanofluid has many applications in industry such as lubricants, coolants, heat exchanger, and micro-channel heat sinks^7^, various biomedical applications of nanofluid are magnetic cell operation, drug delivery, hyperthermia and constant enhancement in Magnetic Resonance Imaging (MRI)^8^. Unlike water-based nanofluids, in the literature; very few researches have been investigated regarding oil-based nanofluid. There are compelling needs in many industrial fields to develop oil-based heat transfer fluids with significantly higher thermal conductivity for energy-efficient heat exchangers. Diathermic oil finds applications in renewable energy and cooling systems. For example, it is used in solar thermodynamic or biomass plants, where high efficiency, compact volumes and high energy fluxes are required. Beside the above applications, diathermic oils are very important in those applications where high temperatures are reached or where the use of water or vapor is not suitable. In Mechanical Engineering and avionics, the colling process of jet and Liquid Rocket Engines (LRE) is a challenging aspect. The heat created during combustion in the rocket engine is contained within the exhaust gases. Most of the heat is expelled along with the gas that contains it, however, heat is transferred to the thrust chamber and at that the chamber and the nozzles experienced high temperature and need to be cooled. For this purpose different techniques are used for the protection of nozzles and chambers. Regenerative cooling is one of the typical technique adopted for the coolant of nozzles and chambers. Thermophysical properties of the fluids have an important role in controlling the cooling performance. In the case of a semi-cryogenic engine, kerosene, the fuel, is being used as a regenerative coolant^9^. Therefore an improvement in thermo-physical properties of diathermic oil, by using of nanoparticles, can increase the performance of the systems^10^. Many efforts have been focused on the oil-based nanofluids. Transformer oil, mineral oil, silicone oil, hydrocarbon fuels, and some organic solutions are used as the base fluids for studying nanofluids. Some interesting works regarding oil-based nanofluids are listed in the refs^11–13^.

Thermo-physical properties change with the change of shape, size, types, base fluid and volume fraction of nanoparticles. Therefore, keeping that in mind, some questions still need to be answered that which nanoparticle is suitable for specified base fluid and which shapes of nanoparticles have more tendency to augment the thermal conductivity and heat transfer rate. A number of research of theoretical and experimental side on nanofluids containing different shapes of nanoparticles very rare in the literature. Hamilton and Crosser^14^ noted enough enhancements in the effective thermal conductivities due to particle shapes. Timofeeva *et al*.^15^ studied experimental and theoretical model of *Al*_2_*O*_3_ nanofluids with different shapes of nanoparticles. Moreover, they considered a base fluid mixture of water and ethylene glycol (EG) and discussed the effect of different shapes of *Al*_2_*O*_3_. Aaiza *et al*.^16^ analyzed heat transfer in MHD mixed convection flow of a ferrofluid along a vertical channel. In another study, Aaiza *et al*.^17^ theoretically investigated energy transfer in MHD mixed convection flow of nanofluids containing different shapes of nanoparticles inside a channel filled with saturated porous medium. Ellahi *et al*.^18^ studied analytically the effect of different shapes of nanoparticles in HEF-7100 using entropy generation method. They found that in HEF-7100 nanofluids, sphere and needle shape nanoparticles has less heat transfer rate than dish shape of nanoparticles. Khan^19^ discussed the effect of the different shapes of MoS_2_ in water and shown graphically that platelet and cylinder shapes have the highest thermal conductivity. Recently Ali *et al*.^20^ investigated the effect of MoS_2_ in engine oil. They concluded that by suspending MoS_2_ nanoparticles can augment the heat transfer rate of engine oil up to 6.35%. Some other important studies on nanofluids are given in^21–24^.

In fluid mechanics, the fractional derivatives approach is widely used to study rheological properties of fluids. Recently, they have been used as an effective tool across physical situations. The most used fractional derivatives are Caputo and Riemann-Liouville fractional derivative operators. Caputo and Fabrizio have recently introduced a new definition of the fractional derivatives with an exponential kernel without singularities^25^. In the areas such as dynamics, bioengineering, and chemistry fractional calculus produced more reliable mathematical models of physical problems than the standard calculus. Many rheological properties of physical materials can only be analyzed by using fractional derivatives. For instant fractional derivatives are used in bio-rheology, plasma physics, astrophysics, biophysics, thermodynamics, traveling wave solutions, optics and electromagnetism^26^.

The literature survey indicates that in the field of nanofluids, carbon nanotubes (CNTs), copper (Cu) and aluminum oxide (*Al*_2_*O*_3_) etc. are common nanoparticles which have been studied from past decades. Most of the researchers are interested in the spherical shape of nanoparticles but they are limited in terms of applications and significance. Due to this reason non-spherical shaped nanoparticles are chosen in this study. More exactly, the present work aims to investigate the effect of four different shapes of MoS_2_ nanoparticles namely cylinder, platelet, blade and brick. To the best of author knowledge, studies on different shapes of MoS_2_ nanoparticles contained in EO and kerosene oil KO as the base fluids are not reported yet. The MoS_2_ nanoparticle is chosen in this work because of its notable and promising applications in many areas especially in two-dimensional electronic devices such as Field Effect Transistors (FETs). Moreover the structure of MoS_2_ is much similar to graphene, it has a single and multilayer sheet with large bandgap structure and furthermore, thermo-physical properties of MoS_2_ nanofluids such as thermal conductivity and heat capacity as well as lubrication abilities also can be used in mechanical applications^27,28^. Therefore in the present work, for the first time, fractional-order Brinkman-type EO-KO based MoS_2_ nanofluid in a rotating frame with Hall effect is investigated. The problem is modeled in terms of partial differential equations and fractional-order in time (Caputo-Fabrizio) approach has been used. Exact solutions are luxury to obtain. Exact solutions provide a bench mark for numerical and experimental solvers. Graphical results for velocity field and temperature distributions are displayed for various parameters of interest and discussed in details.

## Mathematical Formulation

**Figure 1:**
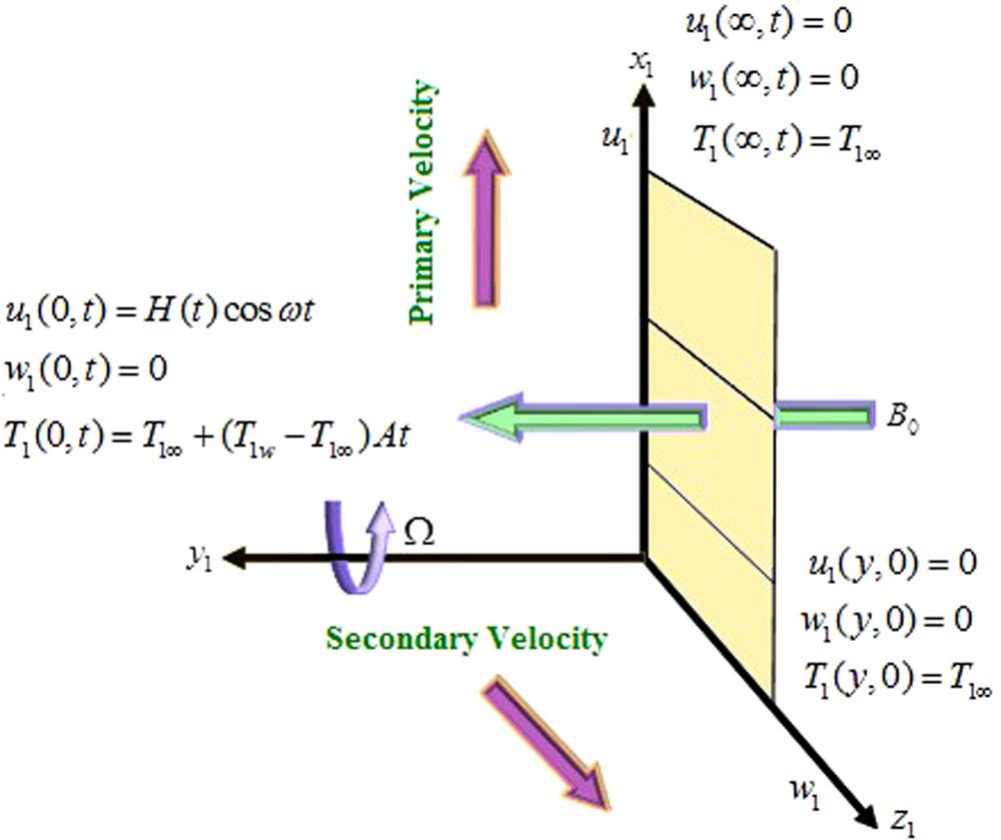
Physical model of the problem.

A three dimension (3-D) fluid flow has been considered in the present work. The fluid flow is considered to be in the *x*_1_- direction, taken along the length of the plate in the upward direction and *y*_1_- axis is normal to it. The fluid is permeated by the uniform transverse magnetic field *B*_0_ in the direction parallel to *y*_1_- axis. Both the fluid and plate rotates as a rigid body rotation with constant angular velocity Ω about *y*_1_- axis. The flow is along *x*_1_- axis and fluid occupies the space *y*_1_ ≥ 0. At the time *t* ≤ 0, both the fluid and plate are at rest and maintained at a uniform temperature *T*_1∞_. Thereafter, i.e. at time *t* > 0, plate starts oscillation with constant amplitude *U*_0_, in *x*_1_− direction. Instantly, the temperature of the plate is raised from *T*_1∞_ to *T*_1∞_ + (*T*_1*w*_ − *T*_1∞_)*At* i.e. at the time *t* > *t*_0_, the plate is preserved at a uniform temperature *T*_1∞_.The plate is considered to be of infinite extent in *x*_1_ and *z*_1_ directions so all physical quantities except pressure is the functions of *y* and *t* only. The geometry of the problem is shown in Fig. 1.

Under the above assumptions, the governing equations for MHD convective flow of fractional-order Brinkman-Type nanofluid in a rotating frame with Hall effect, taking thermal radiation into account and under Boussinesq’s approximation, are given by^12,20^:

1 $$\frac{\partial {u}_{1}}{\partial {t}_{1}}+\beta {u}_{1}-2{\rm{\Omega }}{w}_{1}=\frac{{\mu }_{nf}}{{\rho }_{nf}}\frac{{\partial }^{2}{u}_{1}}{\partial {y}_{1}^{2}}-\frac{{\sigma }_{nf}{B}_{0}^{2}({u}_{1}+m{w}_{1})}{{\rho }_{nf}(1+{m}^{2})}+g{\beta }_{nf}({T}_{1w}-{T}_{1\infty }),$$

2 $$\frac{\partial {w}_{1}}{\partial {t}_{1}}+\beta {w}_{1}+2{\rm{\Omega }}{u}_{1}=\frac{{\mu }_{nf}}{{\rho }_{nf}}\frac{{\partial }^{2}{w}_{1}}{\partial {y}_{1}^{2}}+\frac{{\sigma }_{nf}{B}_{0}^{2}(m{u}_{1}-{w}_{1})}{{\rho }_{nf}(1+{m}^{2})},$$

3 $${(\rho {c}_{p})}_{nf}\frac{\partial {T}_{1}}{\partial {t}_{1}}={k}_{nf}\frac{{\partial }^{2}{T}_{1}}{\partial {y}_{1}^{2}}-\frac{\partial {q}_{1r}}{\partial {y}_{1}},$$

subject to the following initial and boundary conditions:

4 $$\begin{array}{lll}{u}_{1}(y,\,0)=0, & {u}_{1}(0,\,t)=H(t){U}_{0}\,\cos \,\omega t, & {u}_{1}(\infty ,\,t)=0\\ {w}_{1}(y,\,0)=0, & {w}_{1}(0,\,t)=0 & {w}_{1}(\infty ,\,t)=0\\ {T}_{1}(y,\,0)={T}_{1\infty }, & {T}_{1}(0,\,t)={T}_{1\infty }+({T}_{1w}-{T}_{1\infty })At, & {T}_{1}(\infty ,\,t)={T}_{1\infty }\end{array}\},$$

where *H*(*t*) is the Heaviside step function, *A* is the constant with dimension *s*^−1^ and shows the amplitude, *u*_1_, *w*_1_, *β*, Ω, *B*_*o*_, *m*, *T*_1*w*_, *T*_1∞_, *g* and *q*_1*r*_ are the component of nanofluid velocity in *x*_1_-direction, nanofluid velocity in *z*_1_- direction, Brinkman parameter, rotation parameter, magnetic field, Hall current parameter, wall temperature of the plate, ambient temperature, acceleration due to gravity and radiative heat flux respectively, on the other hand *μ*_*nf*_, *ρ*_*nf*_, *σ*_*nf*_, *β*_*nf*_, (*ρc*_*p*_)_*nf*_ and *k*_*nf*_ are presenting the dynamic viscosity, density, electrical conductivity, thermal expansion, heat capacity and thermal conductivity of the nanofluid.

In this work, Hamilton and Crosser model^14^ of thermal conductivity *k*_*nf*_ and dynamic viscosity *μ*_*nf*_ are used. According to this model

5 $${\mu }_{nf}={\mu }_{f}(1+a\varphi +b\varphi ),$$

6 $$\frac{{k}_{nf}}{{k}_{f}}=\frac{{k}_{s}+(n-1){k}_{f}+(n-1)({k}_{s}-{k}_{f})\varphi }{{k}_{s}+(n-1){k}_{f}-({k}_{s}-{k}_{f})\varphi },$$

where *ϕ* is the volume fraction of the nanoparticles and *a* and *b* are shaped constants defined in Table 1. The empirical shape factor *n* defined in Equation. (6) is equal to $n=\frac{3}{\psi }$, where *ψ* is the sphericity. In Hamilton and Crosser model, sphericity is the ratio of surface area of the sphere to the surface area of real particles with equal volumes. The values of sphericity for different shapes of nanoparticles and thermophysical properties are given in Tables 2 and 3, respectively.

**Table 1:** Constants *a* and *b* empirical shape factors^19^.

Model	Platelet	Blade	Cylinder	Brick
*a*	37.1	14.6	13.5	1.9
*b*	6 12.6	123.3	904.4	471.4

**Table 2:** Sphericity *Ψ* for different shapes nanoparticles^19^.

Model	Platelet	Blade	Cylinder	Brick
*ψ*	0.52	0.36	0.62	0.81

**Table 3:** Thermophysical properties of EO and Molybdenum Disulphide^19,22^.

	*ρ* (Kgm^−3^)	*c*_*p*_ (JKg^−1^K^1^)	k (Wm^−1^K^−1^)	*β* × 10^−5^ (K^−1^)	*σ* (s/m)
Engine Oil	863	2048	0.1404	0.00007	55 × 10^−6^
Kerosene Oil	783	2090	0.15	0.00099	21 × 10^−6^
MoS_2_	5.06 × 10^3^	397.21	904.4	2.8424	2.09 × 10^−4^

For the nanofluids, the expressions of *ρ*_*nf*_,*σ*_*nf*_, (*ρβ*)_*nf*_and(*ρc*_*p*_)_*nf*_ are given as^19^:

7 $$\begin{array}{ll}{\rho }_{nf}=(1-\varphi ){\rho }_{f}+\varphi {\rho }_{s}, & {(\rho \beta )}_{nf}=(1-\varphi ){(\rho \beta )}_{f}+\varphi {(\rho \beta )}_{s},\\ {(\rho {c}_{p})}_{nf}=(1-\varphi ){(\rho {c}_{p})}_{f}+\varphi {(\rho {c}_{p})}_{s}, & \frac{{k}_{nf}}{{k}_{f}}={\lambda }_{f},\\ {\sigma }_{nf}={\sigma }_{f}[1+\frac{3(\sigma -1)\varphi }{(\sigma +2)-(\sigma -1)\varphi }], & \sigma =\frac{{\sigma }_{s}}{{\sigma }_{f}}.\end{array}\},$$

We adopt the Roseland’s approximation for radiative heat flux *q*_1*r*_:

8 $${q}_{1r}=\frac{-4{\sigma }^{\ast }}{3{k}^{\ast }}\frac{\partial {T}_{1}^{4}}{\partial {y}_{1}},$$

where σ^*^ is the Stefan-Boltzmann constant and k^*^ is the mean absorption coefficient. The difference between fluid temperature *T*_1_ and free stream temperature *T*_1∞_ is very small so, while applying Taylor series, second and higher order terms are neglected:

9 $${T}_{1}^{4}=4{T}_{1\infty }{T}_{1}^{3}-3{T}_{1\infty }^{4}.$$

Substituting equation (9) into equation (8) and differentiating with respect to *y*_1_, we get:

10 $$\frac{\partial {q}_{1r}}{\partial {y}_{1}}=-\,\frac{16{\sigma }^{\ast }}{3{k}^{\ast }}{T}_{1\infty }^{3}\frac{{\partial }^{2}{T}_{1}}{\partial {y}_{1}^{2}}.$$

Using equation (10) in equation (3), we get:

11 $${(\rho {c}_{p})}_{nf}\frac{\partial {T}_{1}}{\partial {t}_{1}}={k}_{nf}\frac{{\partial }^{2}{T}_{1}}{\partial {y}_{1}^{2}}+\frac{16{\sigma }^{\ast }}{3{k}^{\ast }}{T}_{1\infty }^{3}\frac{{\partial }^{2}{T}_{1}}{\partial {y}_{1}^{2}}.$$

Introducing the following dimensionless variables

$$u=\frac{{u}_{1}}{{u}_{0}},\,y=\frac{{u}_{0}}{\nu }{y}_{1},\,t=\frac{{u}_{0}^{2}}{\nu }{t}_{1},\,w=\frac{{w}_{1}}{{u}_{0}},\,T=\frac{{T}_{1}-{T}_{1\infty }}{{T}_{1w}-{T}_{1\infty }},$$

into equations (1), (2) and (11), we get the following system of dimensionless equations:

12 $$\frac{\partial u}{\partial t}+{\beta }_{1}u-2\eta w={a}_{1}\frac{{\partial }^{2}u}{\partial {y}^{2}}-{M}_{0}(\frac{u+mw}{1+{m}^{2}})+G{r}_{o}T,$$

13 $$\frac{\partial w}{\partial t}+{\beta }_{1}w+2\eta u={a}_{1}\frac{{\partial }^{2}w}{\partial {y}^{2}}+{M}_{0}(\frac{mu-w}{1+{m}^{2}}),$$

14 $$\frac{\partial T}{\partial t}=\frac{1}{{b}_{1}}\frac{{\partial }^{2}T}{\partial {y}^{2}},$$

subject to the following dimensionless initial and boundary conditions:

15 $$\begin{array}{lll}u(y,\,0)=0, & u(0,\,t)=H(t)\cos \,\omega t, & u(\infty ,\,t)=0\\ w(y,\,0)=0, & w(0,\,t)=0, & w(\infty ,\,t)=0\\ T(y,\,0)=0, & T(0,\,t)=t, & T(\infty ,\,t)=0\end{array}\},$$

where

16 $$\begin{array}{rcl}{\beta }_{1} & = & \tfrac{\beta \nu }{{U}_{0}^{2}},\,\eta =\tfrac{{\rm{\Omega }}\nu }{{U}_{0}^{2}},\,{\varphi }_{1}=(1+a\varphi +b{\varphi }^{2}),\,{\phi }_{2}=(1-\varphi )+\varphi \tfrac{{\rho }_{s}}{{\rho }_{f}},\,{\varphi }_{3}=1+\tfrac{3(\sigma -1)\varphi }{(\sigma +2)-(\sigma -1)\varphi },\\ {\phi }_{4} & = & (1-\varphi )+\varphi \tfrac{{(\rho \beta )}_{s}}{{(\rho \beta )}_{f}},\,{\phi }_{5}=(1-\varphi )+\varphi \tfrac{{(\rho {c}_{p})}_{s}}{{(\rho {c}_{p})}_{f}},\,{a}_{1}=\tfrac{{\varphi }_{1}}{{\varphi }_{2}},\,{a}_{2}=\tfrac{{\varphi }_{3}}{{\varphi }_{2}},\,{a}_{3}=\tfrac{{\varphi }_{4}}{{\varphi }_{2}},\\ Gr & = & \tfrac{g{\beta }_{Tf}\nu ({T}_{1}-{T}_{1\infty })}{{U}_{0}^{3}},\,G{r}_{0}=Gr{a}_{3},\,M=\tfrac{{\sigma }_{f}\nu {B}_{0}^{2}}{{\rho }_{f}{U}_{0}^{2}},\,{M}_{0}=M{a}_{2},\,{\lambda }_{f}=\tfrac{{k}_{nf}}{{k}_{f}}\\ {\rm{\Pr }} & = & \tfrac{{(\mu {c}_{p})}_{f}}{{k}_{f}},\,Nr=\tfrac{16{\sigma }^{\ast }{T}_{1\infty }^{3}}{3{k}^{\ast }{k}_{f}},\,{b}_{1}=\tfrac{\Pr {\varphi }_{5}}{{\lambda }_{nf}(1+Nr)}\end{array}\},$$

where *β*_1_ is the dimensionless Brinkman parameter, *η* is the dimensionless rotation parameter, *ϕ*_*i*_(*i* = 1, 2, 3, 4, 5) represent the functions which depend on the thermo-physical properties of the base fluid and nanoparticles, *Gr* is the Grashof number, *M* is the dimensionless magnetic parameter, Pr is the prandtl number and *Nr* is the radiation parameter. The equations (12–14) along with the initial and boundary conditions (15) representing fluid flow, to better understand the flow features, equations (12 and 13) can be expressed in more suitable form as:

17 $$\frac{\partial F(y,\,t)}{\partial t}={a}_{1}\frac{{\partial }^{2}F(y,\,t)}{\partial {y}^{2}}-\lambda F(y,\,t)+G{r}_{0}T,$$

where *F* = *u* + *wi* is a complex velocity and $\lambda ={M}_{0}\frac{(1-mi)}{1+{m}^{2}}+{\beta }_{1}+2{k}^{2}i$ is a constant.

In order to generalized the classical model, $\frac{\partial (y,t)}{\partial t}$ is replace by ${D}_{t}^{\alpha }(y,t)$, equations (14 and 17) become:

18 $${{D}_{t}}^{\alpha }F(y,\,t)={a}_{1}\frac{{\partial }^{2}F(y,t)}{\partial {y}^{2}}-\lambda F(y,\,t)+G{r}_{0}T.$$

19 $${{D}_{t}}^{\alpha }T(y,\,t)=\frac{1}{{b}_{1}}\frac{{\partial }^{2}T(y,\,t)}{\partial {y}^{2}},$$

associated to the following dimensionless initial and boundary conditions are:

20 $$\begin{array}{ll}F(y,\,0)=0, & T(y,\,0)=0\\ F(0,\,t)=H(t)\cos \,\omega t, & T(0,\,t)=t\\ F(\infty ,\,t)=0, & T(\infty ,\,t)=0\end{array}\}.$$

Here ${{D}_{t}}^{\alpha }f(t)$ is known as Caputo–Fabrizio time-fractional operator of order *α* defined by:

21 $${{D}_{t}}^{\alpha }f(y,\,t)=\frac{M(\alpha )}{(1-\alpha )}{\int }_{a}^{t}{f}^{\text{'}}(y,\,t)\exp [\frac{\alpha (t-\tau )}{1-\alpha }]d\tau ,$$

where *M*(*α*) is the normalization function such that *M*(0) = *M*(1) = 1.

## Exact Solution

To evaluate exact solutions for velocity and temperature distribution, the fractional Laplace transformation is utilized here.

### Calculation of temperature distribution

Applying Laplace transform to equation (19) and using the corresponding initial and boundary conditions from equation (20), we get:

22 $$\overline{T}(y,\,s)=\frac{1}{{s}^{2}}\exp (-y\sqrt{\frac{{b}_{2}s}{s+{a}_{5}}}).$$

Equation (22) can be written in equivalent but more simplified form as:

23 $$\overline{T}(y,\,s)=\frac{1}{s}\overline{\chi }(y,\,s,\,0,\,{b}_{2},\,0,\,{a}_{5}).$$

Upon taking the inverse Laplace transformation of equation (23), we get:

24 $$T(y,\,t)={\int }_{0}^{t}\chi (y,\,u,\,0,\,{b}_{2},\,0,\,{a}_{5})du,$$

where

$$\overline{\chi }(y,\,s,\,\lambda ,\,a,\,b,\,c)=\frac{1}{s-\lambda }\exp (-y\sqrt{\frac{as+b}{s+c}}),$$

and

25 $$\begin{array}{rcl}\chi (y,\,t,\,\lambda ,\,a,\,b,\,c) & = & \exp (-\,\lambda t-y\sqrt{a})-\frac{y\sqrt{b-ac}1}{2\sqrt{\pi }}\\  &  & \times {\int }_{0}^{\infty }{\int }_{0}^{t}\frac{{e}^{-\lambda t}}{\sqrt{t}}\exp (\lambda t-ct-\frac{{y}^{2}}{4u}-au){I}_{1}(2\sqrt{(b-ac)ut})dtdu.\end{array}$$

### Calculation of velocity distribution

Applying the Laplace transform to equation (18) and using corresponding initial conditions from equation (20), we get:

26 $$\begin{array}{c}\begin{array}{rcl}\overline{F}(y,\,s) & = & \frac{1}{2(s+i\omega )}\exp (-y\sqrt{\frac{{b}_{3}s+{b}_{4}}{s+{a}_{5}}})+\frac{1}{2(s-i\omega )}\exp (-y\sqrt{\frac{{b}_{3}s+{b}_{4}}{s+{a}_{5}}})\\  &  & +\,\frac{{\Re }_{0}}{(s-{b}_{6})}\exp (-y\sqrt{\frac{{b}_{3}s+{b}_{4}}{s+{a}_{5}}})-\frac{{\Re }_{1}}{{s}^{2}}\exp (-y\sqrt{\frac{{b}_{3}s+{b}_{4}}{s+{a}_{5}}})\\  &  & \,-\frac{{\Re }_{0}}{s}\exp (-y\sqrt{\frac{{b}_{3}s+{b}_{4}}{s+{a}_{5}}})+\frac{{\Re }_{0}}{s-{b}_{6}}\exp (-y\sqrt{\frac{{b}_{2}s}{s+{a}_{5}}})\\  &  & \,-\frac{{\Re }_{1}}{{s}^{2}}\exp (-y\sqrt{\frac{{b}_{2}s}{s+{a}_{5}}})-\frac{{\Re }_{0}}{s}\exp (-y\sqrt{\frac{{b}_{2}s}{s+{a}_{5}}}),\end{array}\end{array}$$

here

$$\begin{array}{rcl}{a}_{4} & = & \frac{1}{1-\alpha },\,{a}_{5}=\alpha {a}_{4},\,{a}_{6}=\frac{{a}_{4}}{{a}_{1}},\,{\lambda }_{1}=\frac{\lambda }{{a}_{1}},\,{b}_{2}={a}_{4}{b}_{1},\,{b}_{3}={a}_{6}+{\lambda }_{1},\,{b}_{4}={\lambda }_{1}{a}_{5},\\ {b}_{5} & = & {b}_{2}-{b}_{3},\,{b}_{6}=\frac{{b}_{4}}{{b}_{5}},\,G{r}_{1}=\frac{G{r}_{0}}{{a}_{1}},\,G{r}_{2}=\frac{G{r}_{1}}{{b}_{5}},\,{\Re }_{0}=\frac{G{r}_{2}}{{{b}^{2}}_{6}},\,{\Re }_{1}=\frac{G{r}_{2}}{{b}_{6}}\end{array}$$

For the convenience in the inversion of Laplace transformation, equation (26) takes the following equivalent form:

27 $$\begin{array}{rcl}\overline{F}(y,\,s) & = & \frac{1}{2}\overline{\chi }(y,s,i\omega ,{b}_{3},{b}_{4},{a}_{5})+\frac{1}{2}\overline{\chi }(y,s,-\,i\omega ,{b}_{3},{b}_{4},{a}_{5})\\  &  & +{\Re }_{0}\overline{\chi }(y,s,{b}_{6},{b}_{3},{b}_{4},{a}_{5})-\frac{{\Re }_{1}}{s}\overline{\chi }(y,s,0,{b}_{3},{b}_{4},{a}_{5})-{\Re }_{0}\overline{\chi }(y,s,0,{b}_{3},{b}_{4},{a}_{5})\\  &  & +{\Re }_{0}\overline{\chi }(y,s,{b}_{6},{b}_{2},0,{a}_{5})-\frac{{\Re }_{1}}{s}\overline{\chi }(y,s,0,{b}_{2},0,{a}_{5})-{\Re }_{0}\overline{\chi }(y,s,0,{b}_{2},0,{a}_{5}).\end{array}$$

Equation (27) is inverted as:

28 $$\begin{array}{rcl}F(y,t) & = & \frac{1}{2}\chi (y,t,i\omega ,{b}_{3},{b}_{4},{a}_{5})+\frac{1}{2}\chi (y,t,-i\omega ,{b}_{3},{b}_{4},{a}_{5})\\  &  & +{\Re }_{0}\chi (y,t,{b}_{6},{b}_{3},{b}_{4},{a}_{5})-{\Re }_{1}{\int }_{0}^{t}\chi (y,u,0,{b}_{3},{b}_{4},{a}_{5})du\\  &  & -{\Re }_{0}\chi (y,t,0,{b}_{3},{b}_{4},{a}_{5})+{\Re }_{0}\chi (y,t,{b}_{6},{b}_{2},0,{a}_{5})\\  &  & -{\Re }_{1}{\int }_{0}^{t}\chi (y,u,0,{b}_{3},0,{a}_{5})du-{\Re }_{0}\overline{\chi }(y,t,0,{b}_{2},0,{a}_{5}),\end{array}$$

## Graphical Discussion

**Figure 2:**
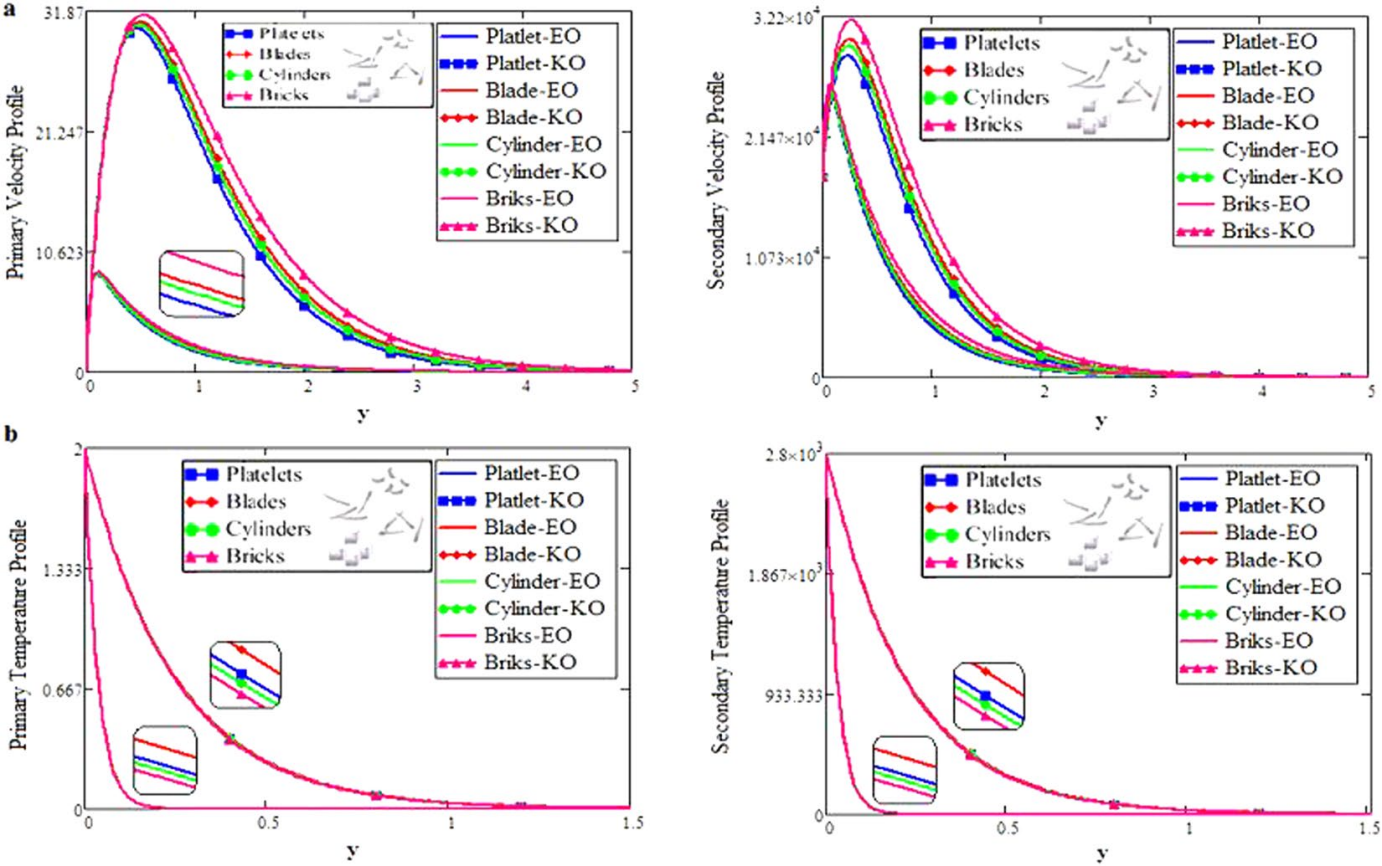
(**a**,**b**) Velocity and Temperature profiles for MoS_2_-EO-KO-based Brinkman-type nanofluid for different shapes of nanoparticles when $\varphi =0.01,Gr=0.5,\Pr =600,m=0.5,\eta =0.5,$ *M* = 3.6, *Nr* = 0.5, *β*_1_ = 0.5 & *α* = 0.2.

The shape effect of MoS_2_ nanoparticles in free convection Brinkman-type Engine and Kerosene oil based nanofluid in a rotating frame with Hall effect is studied. The effect of MHD and thermal radiation is also considered. Hamilton and Crosser model^14^ is used for the expressions of ${\rho }_{nf},\,\rho {\beta }_{nf},\,{(\rho {c}_{p})}_{nf},\,{\sigma }_{nf}\,{\rm{and}}\,\frac{{k}_{nf}}{{k}_{f}}$. In this section, various graphs for different influenced parameters using MATHCAD software, are plotted and illustrated. Figure 2(a,b) are plotted to discuss the effects of different shapes of MoS_2_ nanoparticle on velocity and temperature profiles respectively. The effect of the fractional parameter has been discussed in the figures. It is observed that by increasing the values of *α*, velocity decreases, this behavior indicates that the classical modeled fluid is more viscous than the fractional modeled fluid. The obtained graphical results for the behavior of *α* in present study agrees well with the results mentioned by Muhammad *et al*.^29^, Zafar and Fetecau^30^ and Shaikh *et al*.^31^.

**Figure 3:**
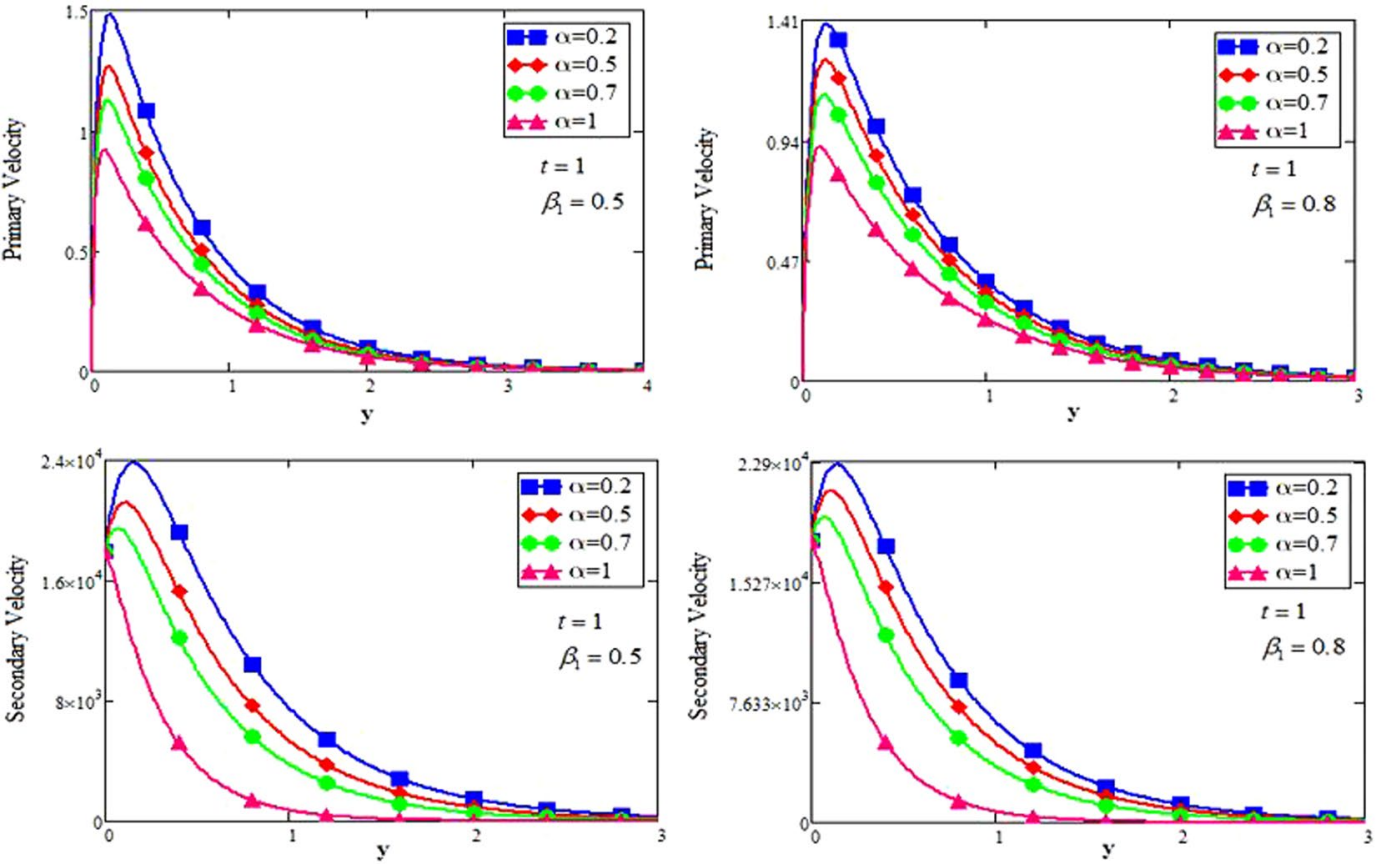
Velocity profile of MoS_2_-EO-based Brinkman-type nanofluid for different values of *β*_1_ when *M* = 3.6, *Nr* = 0.5. $\varphi =0.01,Gr=0.5,\Pr =600,m=0.5\,\& \,\eta =0.5$.

**Figure 4:**
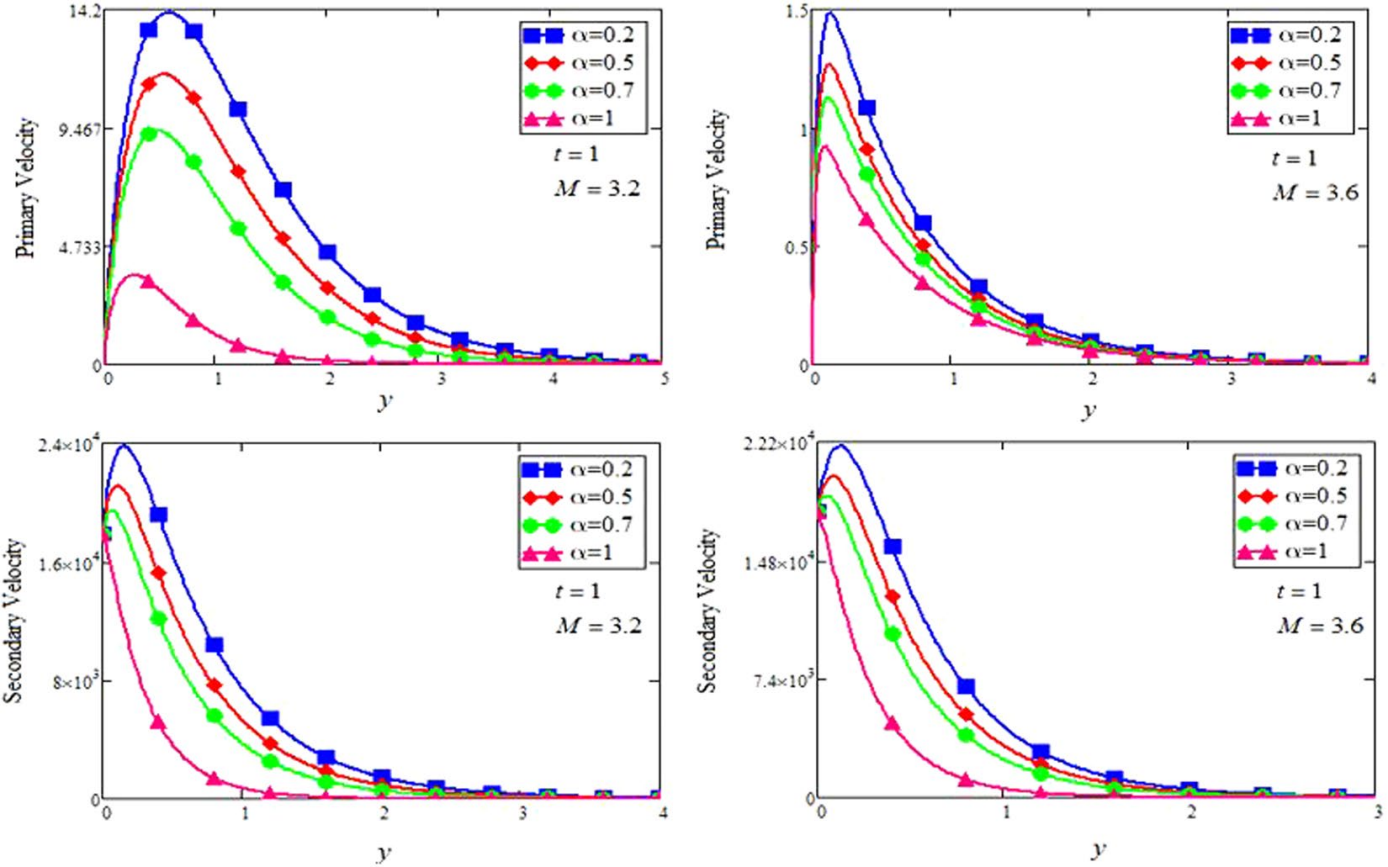
Velocity profile of MoS_2_-EO-based Brinkman-type nanofluid for different values of *M* when *β*_1_ = 0.5, *Nr* = 0.5. $\varphi =0.01,Gr=0.5,\Pr =600,m=0.5\,\& \,\eta =0.5$.

**Figure 5:**
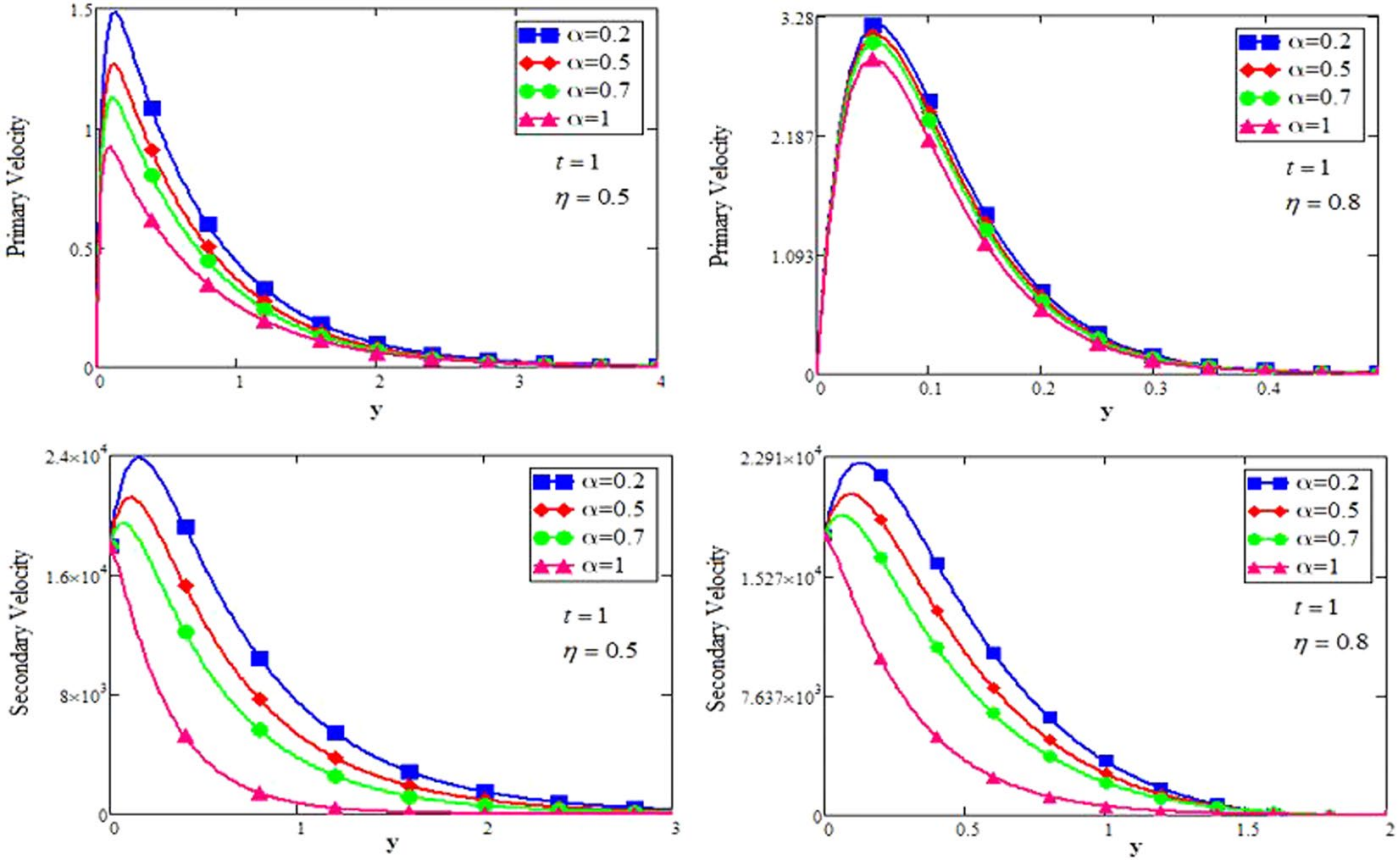
Velocity profile of MoS_2_-EO-based Brinkman-type nanofluid for different values of *η* when $\varphi =0.01,Gr=0.5,\Pr =600,m=0.5,M=3.6,{\beta }_{1}=0.5,Nr=\mathrm{0.5.}$

**Figure 6:**
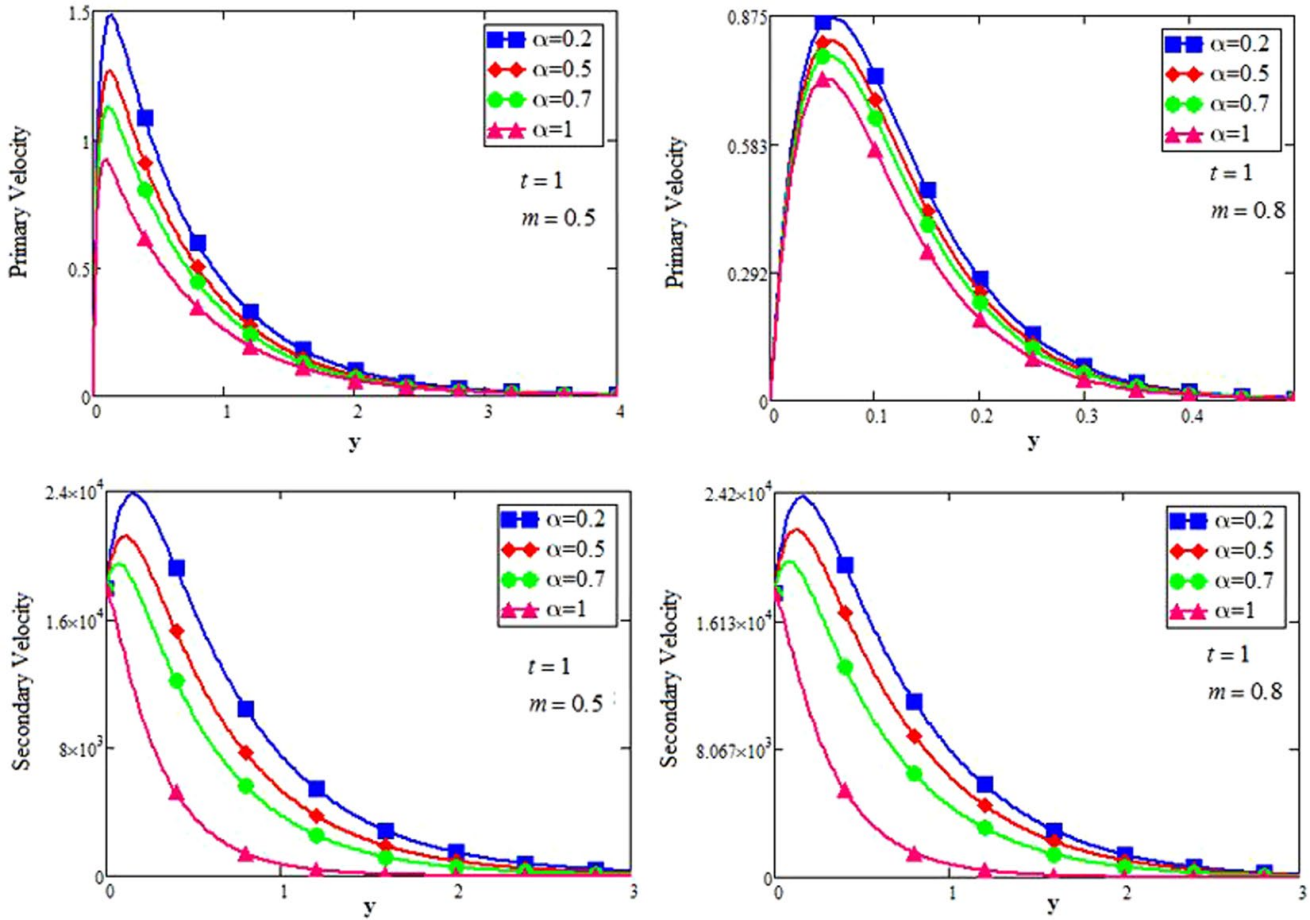
Velocity profile of MoS_2_-EO-based Brinkman-type nanofluid for different values of *m* when $\varphi =0.01,Gr=0.5,\Pr =600,\eta =0.5,M=3.6,{\beta }_{1}=0.5,Nr=\mathrm{0.5.}$

**Figure 7:**
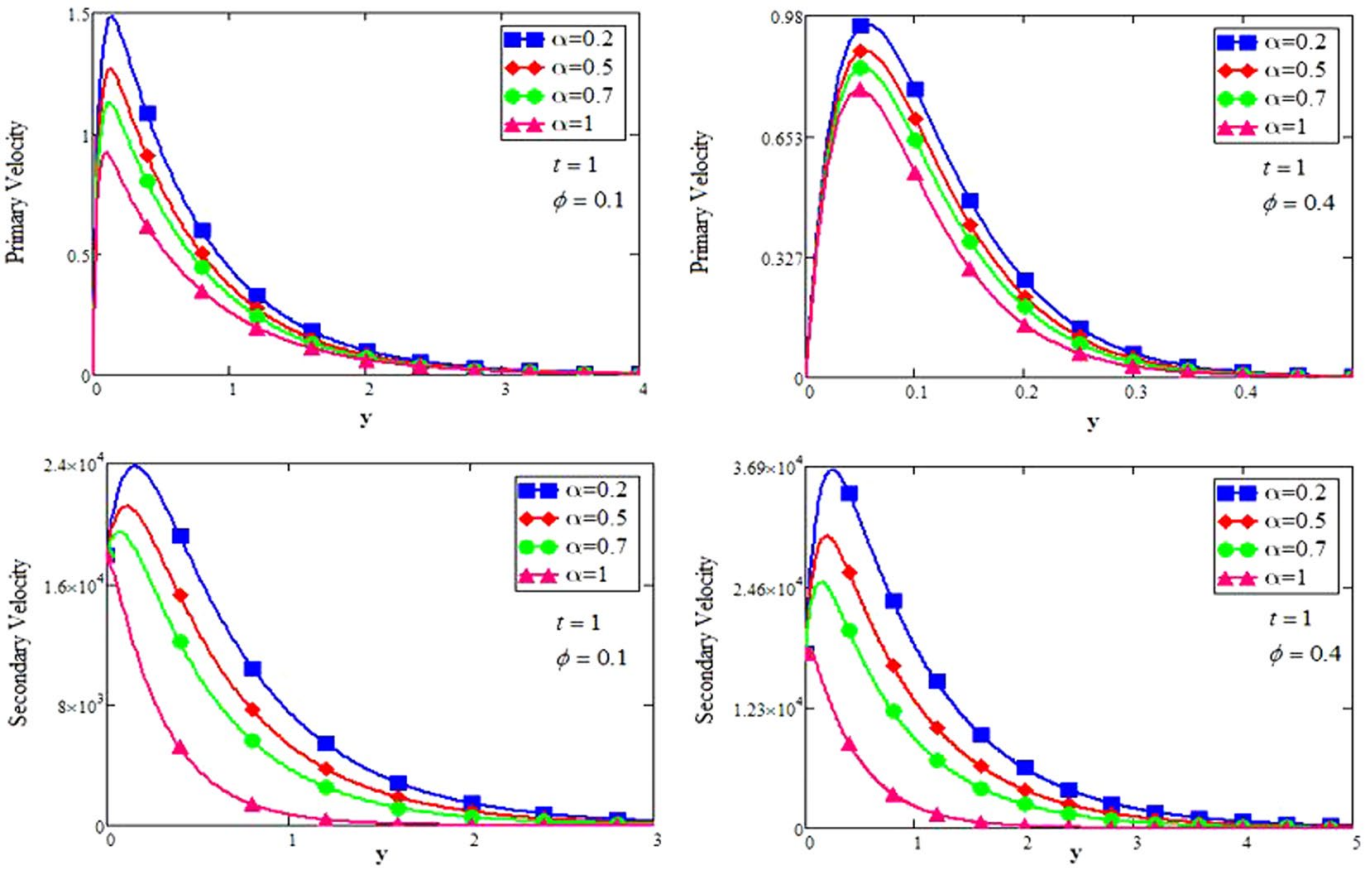
Velocity profile of MoS_2_-EO-based Brinkman-type nanofluid for different values of *ϕ* when $\eta =0.5,Gr=0.5,\Pr =600,m=0.5,M=3.6,{\beta }_{1}=0.5,Nr=\mathrm{0.5.}$

**Figure 8:**
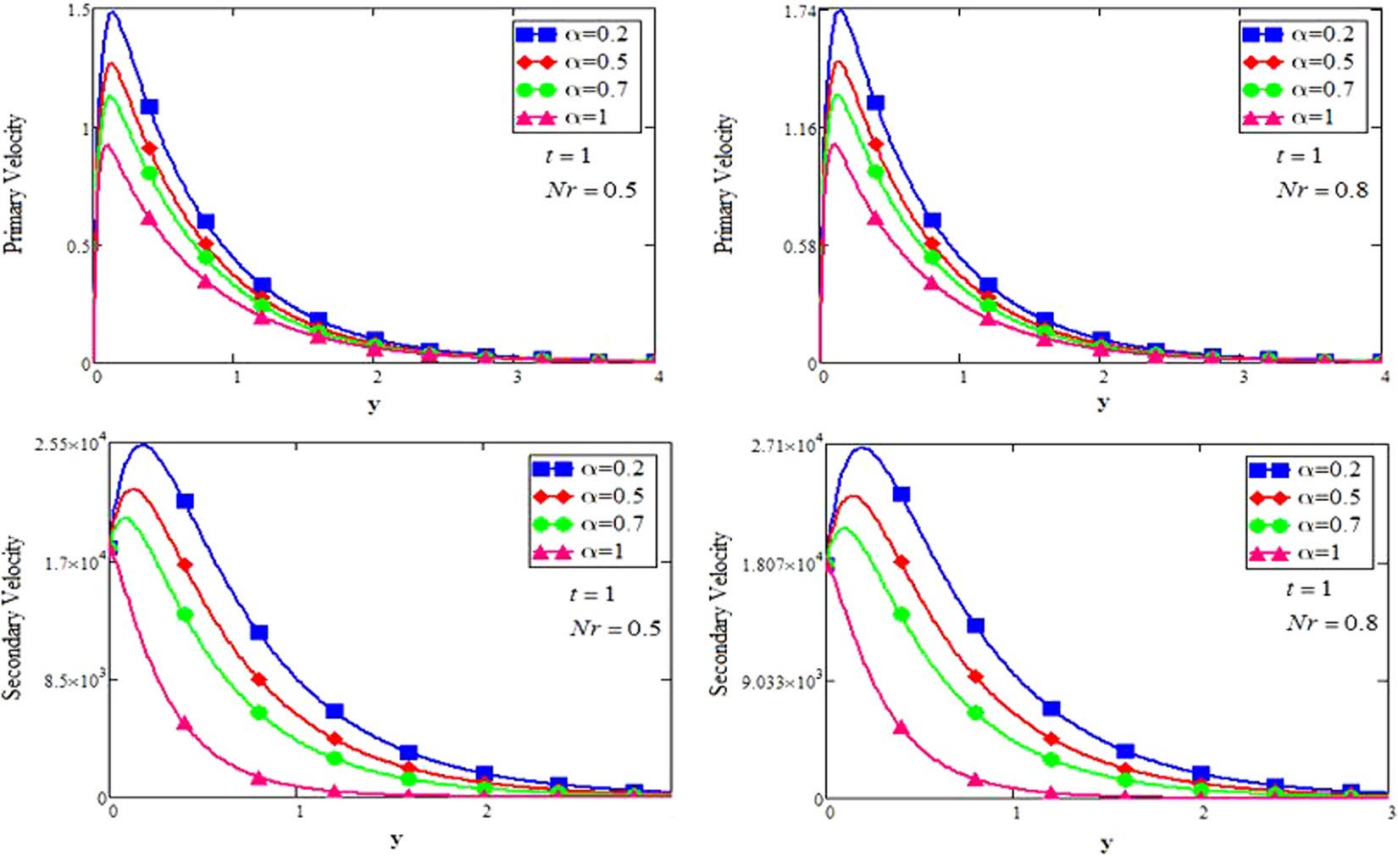
Velocity profile of MoS_2_-EO-based Brinkman-type nanofluid for different values of *Nr* when $\varphi =0.01,m=0.5,\Pr =600,\eta =0.5,M=3.6,{\beta }_{1}=0.5,Gr=\mathrm{0.5.}$

**Figure 9:**
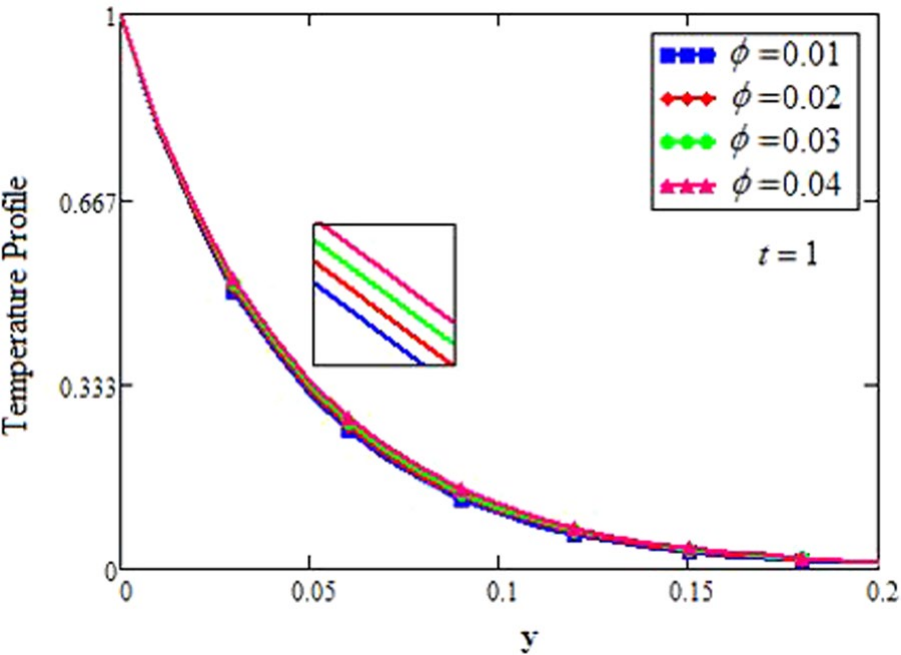
Temperature profile of MoS_2_-EO-based Brinkman-type nanofluid for different values of *ϕ* when $\alpha =0.2,\Pr =600,Nr=0.2$.

**Figure 10:**
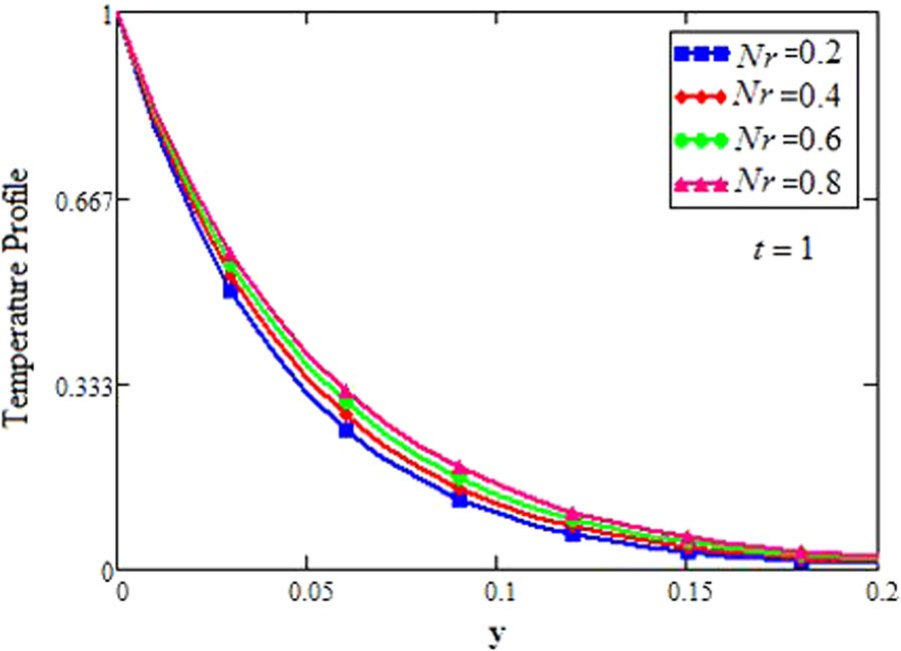
Temperature profile of MoS_2_-EO-based Brinkman-type nanofluid for different values of *Nr* when $\alpha =0.2,\Pr =600,\varphi =0.01$.

The effect for different shapes (blade, brick, cylinder and platelet) of MoS_2_ in engine oil (EO) and kerosene oil (KO) can be seen from Fig. 2(a,b). It can be clearly observed from Fig. 2(a) that MoS_2_ has shown higher velocity in KO as compared to EO. It is also important to notice that Hamilton and Crosser model^14^ for the same *ϕ* is used to investigate the thermal conductivity and viscosity of the EO and KO based nanofluids. This result indicates that EO based nanofluid is more viscous and has higher thermal conductivity followed by KO based nanofluid. It is noticed that when *ϕ* = 0.01, brick shape MoS_2_ nanoparticles have the higher velocity as compare to the blade, cylinder and platelet shapes of the nanoparticles, which consequently shows that platelet shape has the higher viscosity for both the primary and secondary velocity profiles. The present results agree with the experimental work by Temofeeva *et al*.^15^ where they investigated that the platelet shape has the higher viscosity and they found their results identical to Hamilton and Crosser model. The present work also agreed with the recent work by Khan^19^, where he studied the different shapes of MoS_2_ nanoparticle taking water as a base fluid. He highlighted that bricks shape nanoparticle has the highest velocity followed by the blade, cylinder and platelet-shaped nanoparticles when 0.01 ≤ *ϕ* ≤ 0.03. Influence of different shapes of nanoparticles on temperature profile is discussed in Fig. 2(b). It is observed that blade shape of MoS_2_ nanoparticle has the highest temperature followed by platelet, cylinder and brick. It is important to take into consideration that for higher temperature, viscosity decreases. It is clear that the shape of platelet and cylinder have greater viscosity due to which these shapes have a minimum temperature, whereas blade and brick have the highest temperature due to least viscosity. It is also found from the figure that brick shape of nanoparticle has low viscosity. This is due to the shear thinning behavior with temperature. This behavior of different shapes on temperature profile is also studied by Temofeeva^15^, Aiza *et al*.^17^ and Khan^19^. Figure 3 has been plotted to show the influence of Brinkman parameter *β*_1_ on fluid velocity. It is observed for both the primary and secondary velocities that *β*_1_ = 0.5 have greater fluid velocity than *β*_1_ = 0.8. Physically, it is true because Brinkman parameter *β*_1_ is a ratio between drag forces and density, so by increasing *β*_1_ drag forces increased which cause a decrease in velocity which tends to increase the viscosity and consequently enhanced the lubrication properties of engine oil. The same behavior for *β*_1_ has been noticed by Ali *et al*.^32^, which was the first research work on water-based Brinkman-type nanofluid over a vertical plate embedded in a porous medium with variable velocity. Figure 4 is plotted to present the effect of the magnetic parameter *M* on primary and secondary velocity profiles. The results show that by applying high magnetic field strength both the primary and secondary velocities become decreased, this is an obvious result because magnetic field raises the Lorentz forces of an electrically conducted fluid which tends to slow down the motion of the fluid in the boundary layer. The outcomes for different values of *η* can be seen in Fig. 5. It is clear from the figure that rotation has the tendency to accelerate the primary velocity while it has a reverse effect on secondary velocity profile. This happens because the effect of the Coriolis force is dominant near the axis of rotation. Sarkar and Seth^33^ noticed the same behavior for rotation in their work. Figure 6 depicts the effect of Hall current parameter *m* on primary and secondary velocities. The figure illustrated that for *m* = 0.5 the velocity is greater than *m* = 0.8, which shows that Hall current decelerate the primary fluid velocity whereas it has an opposite effect on the secondary fluid velocity, secondary fluid velocity enhanced for higher values of *m*. The present results of Hall current concurred with the results obtained by Sarkar & Seth^33^. The behavior of primary and secondary velocities for different values of nanoparticle volume fraction *ϕ* can be seen in Fig. 7. It is found from the figure that by increasing the values of *ϕ* from 0.01 to 0.04, the fluid becomes more viscous which leads to decrease the primary velocity. A decrease in the velocity means that the viscosity of EO is increased which consequently increase the boiling and freezing point of the EO due to which the lubrication properties of EO become more effective and efficient. The present result for *ϕ* is also experimentally reported by Colla *et al*.^34^. On the other hand, it showed an opposite effect on secondary velocity, secondary velocity is increased by increasing the volume fraction of the nanofluid. Figure 8 highlights the influence of thermal radiation *Nr* on the fluid velocity, which shows that by increasing the values of *Nr* both of the primary and secondary velocities are enhanced. Physically, this means that with the increase of *Nr*, increases the amount of heat energy transfers to the fluid which consequently accelerate the fluid flow. The behavior of *Nr* in the present work is similar to the results reported by Gull *et al*.^17^. They had compared their result with Makinde and Mhone^35^ and found their results similar to them. From Fig. 9 it is found that by taking higher values of volume fraction *ϕ*, the temperature of the fluid is also get increased. Gull *et al*.^17^ also reported the same behavior for *ϕ*, they discussed that temperature of the fluid enhanced for larger values of volume fraction due to the shear thinning behavior. The same behavior of *Nr* on temperature profile has been discussed in Fig. 10. it can be clearly seen from the figure that temperature enhanced for higher values of *Nr*. The effect of *Nr* on the nanofluid temperature is in agreement with its physical behavior to enhance the conduction effect, resulting in the enhancement of the nanofluid temperature in the boundary layer region. The obtained result is agree with the theoretical study of Sharma *et al*.^36^. The effect of different embedded parameters on Nusselt number has been shown in Table 4, it is observed from the table that for higher values of *ϕ*, *α* and *t*, Nusselt number become increased while for *Nr* it shows reverse effect. Impact of volume fraction on Nusselt number discussed in Table 5 it can be seen from the table that for *ϕ* = 0.01 to 0.04, Nusselt number of enhanced from 3.42% to 13.51% respectively. The enhancement of heat transfer rate with different shapes of nanoparticles with different volume fractions has been illustrated in Table 6. It can be observed that blade shaped nanoparticles shown highest variation followed by platelet, cylinder and brick shaped nanoparticles. From the same table it can be noticed that the heat transfer rate of engine oil has been increased by 18.95% with blade-shaped nanoparticles on the other hand 11.48%, 13.51% and 8.95% heat transfer rate is enhanced with cylinder, platelet and brick shaped nanoparticles respectively.

**Table 4:** Impact of various parameter on Nusselt Number.

*ϕ*	*Nr*	*α*	*t*	*Nu*
0.01	0.2	0.2	1	24.334
0.02	0.2	0.2	1	25.131
0.01	**0.4**	0.2	1	22.604
0.01	0.2	**0.4**	1	25.604
0.01	0.2	0.2	**1.5**	35.461

**Table 5:** Impact of Volume Fraction on Nusselt Number and Percent Enhancment.

*ϕ*	*Nr*	*α*	*t*	*Nu*	%
0	0.2	0.2	1	23.53	—
0.01	0.2	0.2	1	24.334	3.42
0.02	0.2	0.2	1	25.131	6.80
0.03	0.2	0.2	1	25.923	10.16
0.04	0.2	0.2	1	26.71	13.51

**Table 6:** Enhancement of heat transfer rate with different shapes of nanoparticles and volume function.

*ϕ*	Cylinder	Platelet	Brick	Blade
0	23.53	23.53	23.53	23.53
0.01	24.209	24.334	24.055	24.676
0.02	24.889	25.131	24.581	25.799
0.03	25.559	25.923	25.108	26.902
0.04	26.232	26.71	25.636	27.989

## Concluding Remarks

The work reported in this paper, aims to investigate the magnetohydrodynamic (MHD) free convection flow of Brinkman-Type Engine oil EO and Kerosene oil KO based nanofluid in a rotating frame. Effect of Hall current and thermal radiation is also considered. The governing equations of momentum and energy are solved for the solutions of velocity (primary and secondary) and temperature profiles by using Caputo-Fabrizio (CF) time fractional derivative. Different non-spherical shapes of Molybdenum disulfide MoS_2_ nanoparticles namely Platelet, Blade, Cylinder and Bricks are suspended in EO and KO. Hamilton and Crosser model of thermal conductivity is used for non-spherical shapes of nanoparticles. The important results which can be found in the present study are following^37,38^:

- The MoS_2_ nanoparticle is more effective in EO as compared to KO.
- The elongated particles (Platelet & Cylinder) inside EO-KO based nanofluids results in higher viscosity due to its structure and hence, the boiling point of the EO-KO based nanofluid is higher than the regular EO and KO fluids. This will intensely increase the heat carrying capacity and lubrication properties of the oils.
- The more viscous will be the fluid the least will be the freezing point. By adding the nanoparticles, the freezing point of the EO and KO could be controlled at a very low temperature.
- The heat transfer rate of EO-based nanofluid with blade-shaped MoS_2_ nanoparticles is 7.87%, 9.64%, 14.33% and 18.95% greater than platelet, cylinder and brick shaped nanoparticles.
- Heat transfer rate of EO based nanofluid with platelet shaped nanoparticles are 3.42%, 6.80%, 10.16% and 13.51% greater as compared to regular fluid for volume fraction *ϕ* = 0.01 to 0.04 respectively.
- Classical modeled fluid is more viscous than the fractional modeled fluid, for higher values of volume fraction *α*.
- The supplementary materials are uploaded separately.

## Electronic supplementary material

Appendix A
