# Supplementary material for: Effects of Different Shaped Nanoparticles on the Performance of Engine-Oil and Kerosene-Oil: A generalized Brinkman-Type Fluid model with Non-Singular Kernel
Source: Sci Rep. 2018 Oct 16;8:15285. doi: 10.1038/s41598-018-33547-z (PMC6191421; doi:10.1038/s41598-018-33547-z)
Supplement: Supplementary file 1 — Appendix A [file 41598_2018_33547_MOESM1_ESM.docx]

**Effects of Different Shaped Nanoparticles on the Performance of Engine-Oil and Kerosene-Oil: A generalized Brinkman-Type Fluid model with Non-Singular Kernel**

Farhad Ali^*,1,2,3^, Aamina^3^, Ilyas Khan^4^, Nadeem Ahmad Sheikh^1,2,3^ and Madeha Gohar^1,2,3^

^1^Computational Analysis Research Group, Ton Duc Thang University, Ho Chi Minh City, Vietnam.

^2^Faculty of Mathematics and Statistics, Ton Duc Thang University, Ho Chi Minh City, Vietnam.

^3^Department of Mathematics, City University of Science and Information Technology, Peshawar, Khyber Pakhtunkhwa, Pakistan.

^4^Basic Engineering Sciences Department, College of Engineering Majmaah University, Majmaah 11952, Saudi Arabia.

*Corresponding Author: [farhad.ali@tdt.edu.vn](mailto:farhad.ali@tdt.edu.vn)

**Appendix: A**

The Laplace transform of the fractional derivative used in equation (21) is given as; [37 & 38]

$$L\left(D_{t}^{\alpha}f(t)\right)=\frac{1}{\left(1-\alpha\right)}\int_{0}^{\infty} \exp\left(-pt\right)\int_{0}^{t} f\left(\tau\right)\exp\left(-\frac{\alpha\left(t-\tau\right)}{1-\alpha}\right) d\tau dt$$

Hence, from the property of Laplace transform of convolution, we have

$$\begin{matrix} L\left[D_{t}^{\alpha}f(t)\right]=\frac{1}{(1-\alpha)}L\left[f(t)\right]L\left[\exp\left(-\frac{\alpha t}{1-\alpha}\right)\right], \\ =\frac{\left[pL\left\{f(t)-f(0)\right\}\right]}{p+\alpha(1-p)}. \end{matrix}$$
